# Development of Subcutaneous SSEA3- or SSEA4-Positive Cell Capture Device

**DOI:** 10.3390/bioengineering11060585

**Published:** 2024-06-08

**Authors:** Yasuhide Nakayama, Ryosuke Iwai

**Affiliations:** 1 Osaka Laboratory, Biotube Co., Ltd., Osaka 565-0842, Japan; 2 Institute of Frontier Science and Technology, Okayama University of Science, Okayama 700-0005, Japan; iwai@ous.ac.jp

**Keywords:** stem cell, in-body tissue architecture, cell capture, cell accumulation, in vivo tissue engineering

## Abstract

Securing high-quality cell sources is important in regenerative medicine. In this study, we developed a device that can accumulate autologous stem cells in the body. When small wire-assembled molds were embedded in the dorsal subcutaneous pouches of beagles for several weeks, collagen-based tissues with minimal inflammation formed inside the molds. At 3 weeks of embedding, the outer areas of the tissues were composed of immature type III collagen with large amounts of cells expressing SSEA3 or SSEA4 markers, in addition to growth factors such as HGF or VEGF. When separated from the tissues by collagenase treatment, approximately four million cells with a proportion of 70% CD90-positive and 20% SSEA3- or SSEA4-positive cells were recovered from the single mold. The cells could differentiate into bone or cartilage cells. The obtained cell-containing tissues are expected to have potential as therapeutic materials or cell sources in regenerative medicine.

## 1. Introduction

In recent years, many studies on regenerative medicine and cell therapy have been conducted for every tissue and organ in the human body, and many clinical studies and trials are ongoing worldwide [1]. Having a sufficient cell source is one of the most important requirements for regenerative medicine, as it is necessary to prepare a large number of high-quality cells to obtain therapeutic effects. Stem cells are certainly attractive cell sources for regenerative medicine, given their high proliferative potential and multi-lineage cell differentiation characteristics. Embryonic stem (ES) cells and induced pluripotent stem (iPS) cells have pluripotency and a high proliferation potential [2,3,4]. However, these stem cells present ethical and safety issues and many barriers that must be overcome prior to clinical application [5].

Stem cells that can be obtained from the adult human body are useful for cell therapies as they can differentiate into tissue-specific cells and produce trophic factors. Autologous stem cells have no risk of immune rejection or tumorigenesis. Mesenchymal stem cells (MSCs) are commonly used in regenerative medicine for the aforementioned reasons [6]. MSCs can be obtained from the bone marrow, fat tissues, synovial membranes, umbilical cord, and placenta et al. [7,8]. MSCs are basically present in all adult organs and tissues in mice [9]. Furthermore, MSCs have been reported to aggregate around blood vessels [10]. However, the presence rate of MSCs in bone marrow is extremely low, at 0.001~0.01% [11]. Therefore, in order to use MSCs for treatment, a culture process is essential. In addition, MSCs have limited growth and differentiation potential, and cellular senescence occurs in in vitro cultures, resulting in decreased MSC function [12,13]. Therefore, it is desirable to obtain a large number of stem cells from living organisms in a highly active state. Stem cells in tissues contribute to wound healing, tissue repair, and regeneration [14,15]. Biological responses may be useful tools in understanding stem cell accumulation and activation. Recent studies have reported that autologous stem cells, such as MSCs and hematopoietic stem cells (HSCs), are present during foreign body reactions [16,17]. These stem cells have been suggested to contribute to tissue formation, including angiogenesis.

We are working to develop an in vivo tissue engineering technique called “in-body tissue architecture” (iBTA) that allows tissue preparation for autologous implantation by temporarily embedding molds [18,19]. This technology is based on biological reactions against embedded foreign materials. The mold has a basic structure in which a plastic mandrel is inserted into a stainless-steel pipe [20]. The pipe has many slits as openings. The surrounding tissues penetrate the molds through the slits and fill the space between the pipe and mandrel with connective tissues, mainly composed of collagen. By using iBTA, we successfully obtained tubular tissues called “biotubes” [18,20] or heart valve-like tissues called “biovalves” [21] without the need for culturing cells.

To complete the tissue formation of biotubes and biovalves, the thin hollow spaces of the respective preparation molds must be completely filled with collagenous connective tissue, which usually takes 1 to 2 months to produce. Recently, in the formation of biotubes using a pipe-shaped mold with slits, we found an accumulation of autologous stem cells expressing pluripotent MSC markers, including CD90 and stage-specific embryonic antigen 4 (SSEA4) [22]. In biotubes, the high-density collagen and stem cells around the slits were clearly separated. Autologous stem cells greatly contribute to the formation of biotubes since they have tissue repair activity. In this study, we aimed to actively collect somatic stem cells expressing SSEA3 or SSEA4 markers in the body. Because the stem cells were gathered in the slit regions in our previous study [22], we planned to develop a caged mold mainly composed of slits with a large space. This study proposes an efficient in vivo stem cell capture device to improve therapeutic techniques in regenerative medicine.

## 2. Materials and Methods

### 2.1. Mold Preparation

**Figure 1:**
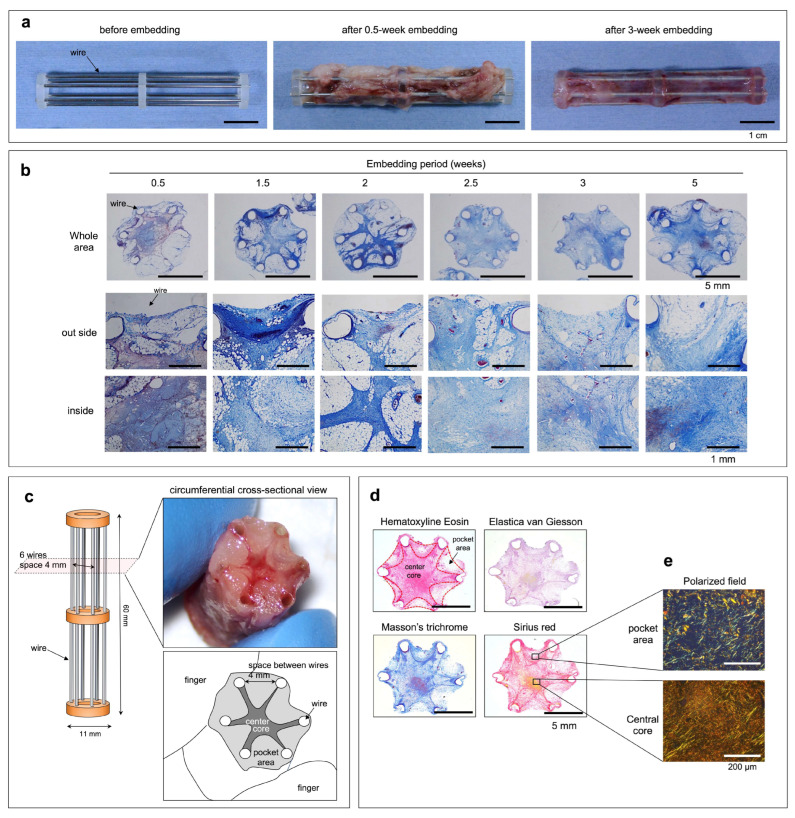
(**a**) The six-wire-framed mold before and after subcutaneous embedding. (**b**) Microscopic cross-sectional photos of the entire, outer, and central side areas of the tissues obtained from the molds during each embedding period after Masson’s trichrome stain. (**c**) The model of the six-wire-framed mold and macroscopic circumferential cross-sectional view of the tissue obtained after 2.5 weeks of embedding. (**d**) Microscopic cross-sectional photos of the tissues obtained after 3 weeks of embedding and H&E, Elastica van Giesson, Masson’s trichrome, or Sirius Red stains. (**e**) Polarized fields at the pocket area or central core of the Sirius Red-stained image.

**Figure 2:**
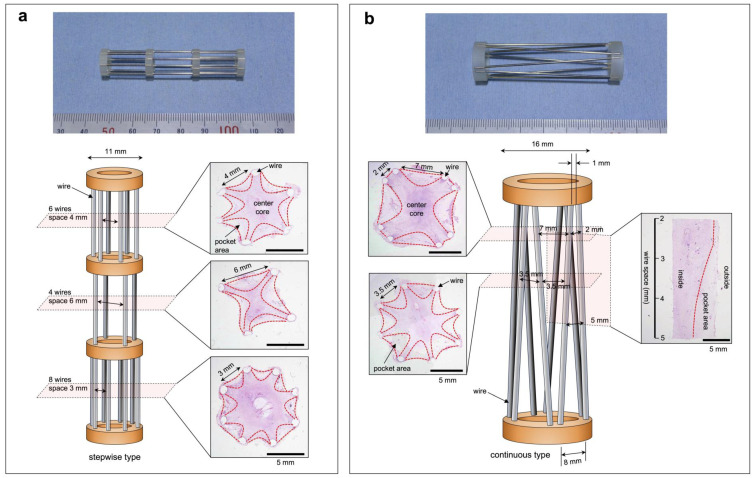
(**a**) Macroscopic view and the model picture of the wire-framed mold with stepwise changes in the wire spacing (3, 4, and 6 mm). Microscopic cross-sectional images of tissue formed inside the mold by 3 weeks of embedding and H&E staining at each wire spacing step. (**b**) Macroscopic view and the model picture of the wire-framed mold with continuous changes in the wire spacing from 1 to 8 mm. Microscopic cross-sectional images of tissue formed inside the mold by 3 weeks of embedding and H&E staining at the wire spacing of 2 and 7 mm, or 3.5 mm. A microscopic longitudinal section of tissue formed inside the mold after H&E staining at a wire spacing of 2 to 5 mm.

Molds were assembled using several stainless steel wires (diameter: 1 mm) and disk-shaped plastic rings made using a 3D digital printer (ProJet 3510 HD Plus; 3D Systems, Rock Hill, SC, USA). Two types of molds with different arrangements of stainless steel wires were prepared (straight or continuous). In the straight type (Figure 1c), six stainless steel wires (length: 60 mm) were arranged in a regular hexagon at 4-mm intervals and fixed with plastic rings (diameter: 10 mm). In the continuous type (Figure 2b), eight stainless steel wires were alternately arranged diagonally with respect to the plastic rings (diameter: 16 mm) such that the distance between the wires changed continuously from 1 to 8 mm. The stepwise type (Figure 2a) was made with 4–8 stainless steel wires (length: 20 mm), which were arranged at equal intervals of 3–6 mm and fixed with plastic rings (diameter: 10 mm).

### 2.2. Mold Embedding

All animal experiments were performed under general anesthesia in accordance with the Guide for the Care and Use of Laboratory Animals published by the United States National Institutes of Health (NIH Publication No. 85-23, revised 1996). The study protocol was approved (No. HD16-009) by the ethics committee of Hamaguchi Lab Plus Inc. (Osaka, Japan).

The preanesthetic medications included ketamine (5 mg/kg), intravenous (IV) solution (0.02 mg/kg), and intramuscular atropine sulfate (0.025 mg/kg). Anesthesia was induced by the IV administration of pentobarbital (15 mg/kg) and maintained with a bolus IV injection of pentobarbital at a quarter or half of the initial dose. The molds were embedded into the dorsal subcutaneous pouches of female beagles (weighing approximately 10 kg each) for up to 5 weeks. After the embedding period, the tissues formed in the molds were harvested, and the connective tissues around the molds were trimmed.

### 2.3. Histological and Immunohistochemical Analysis

The tissues were fixed in 4% paraformaldehyde in phosphate-buffered saline (PBS; pH 7.4) (Wako Pure Chemical Industries, Osaka, Japan), embedded in paraffin, and sectioned into 3–5-µm-thick sections. The sections were stained with hematoxylin and eosin (H&E), Elastica van Gieson (EVG), and Masson’s trichrome (MT). Sirius Red staining was performed to detect the collagen fibers. In brief, deparaffinized and rehydrated sections were stained with a 0.1% Sirius Red solution for 1 h, followed by washing with 0.5% HCl. Sections stained with Sirius Red were observed under a polarized light microscope to distinguish between type I (red or orange) and type III (green) collagen fibers.

For the immunohistochemical analysis, deparaffinized sections were microwaved for 15 min at 150 W in 0.1 M citrate buffer (pH 6.0) (Sigma Aldrich, St. Louis, MO, USA) to retrieve antigens. The sections were then washed twice in distilled water for 10 min, blocked with 1% bovine serum albumin (Wako) in PBS at room temperature for 1 h, and incubated overnight at 4 °C with anti-CD90 mouse monoclonal antibody (1:100; ab123511, Abcam, Cambridge, UK), anti-CD105 mouse monoclonal antibody (1:10; ab156756, Abcam), anti-SSEA4 mouse monoclonal antibody (1:200; ab16287, Abcam), anti-SSEA3 rabbit polyclonal antibody (1:100; bs-375R, Bioss Antibody, Woburn, MA, USA), anti-VEGF mouse monoclonal antibody (1:200; ab1316, Abcam), and anti-HGF rabbit polyclonal antibody (1:100; ab83760, Abcam). After washing twice with distilled water for 10 min, the sections were incubated with Alexa Fluor^®^ 488 goat anti-mouse IgG H&L antibody (1:1000; ab150113, Abcam) or Alexa Fluor^®^ 594 goat anti-rabbit IgG H&L antibody (1:1000; ab150080, Abcam) at room temperature for 2 h. The sections were then washed twice with distilled water for 10 min. ProLong^®^ Gold Antifade Mountant with DAPI (Thermo Fisher, Waltham, MA, USA) was dropped onto the sections, and cover glasses were placed on top. The slides were analyzed using fluorescence microscopy.

### 2.4. Cell Isolation

A collagenase treatment was carried out to harvest cells from the iBTA tissues. After harvesting the molds from the subcutaneous pouches, the iBTA tissues were obtained by trimming the connective tissues around the molds. Tissues with molds were washed in PBS and treated with 0.25% collagenase type 1 solution (Worthington Biochemical, Lakewood, NJ, USA) diluted in DMEM-LG (Wako) at 37 ℃ for 1.5 h under rocking at a rate of 180 rpm. After incubation, the molds and remaining tissues were removed and centrifuged at 1000 rpm for 5 min at 4 °C. The supernatant was removed, and DMEM was added. This was centrifuged at 1000 rpm for 5 min at 4 °C. The supernatant was removed, and 1 mL of Lysing Solution (Beckman Coulter, Brea, CA, USA) was added to remove red blood cells. This was incubated for 10 min at room temperature. After incubation, 10 mL of DMEM containing 10% FBS were added (Biosera, Nuaille, France) and centrifuged at 1000 rpm for 5 min at 4 °C. The supernatant was removed. Banbanker (Nihon Genetics, Tokyo, Japan) was added, and the cells were resuspended, counted, and stored in liquid nitrogen.

### 2.5. Flowcytometry Analysis

To analyze the cell surface antigens, the cryopreserved cells were thawed, washed with PBS, and stained with fluorescein isothiocyanate (FITC)-conjugated anti-CD90 (BioLegend, San Diego, CA, USA) antibody, anti-SSEA4 mouse monoclonal antibody (ab16287, Abcam), Alexa Fluor^®^ 488 goat anti-mouse IgG antibody (ab150113, Abcam), anti-SSEA3 rabbit polyclonal antibody (bs-375R, Bioss), and Alexa Fluor^®^ 488 goat anti-rabbit IgG antibody (ab150077, Abcam). The cells were then analyzed using a flow cytometer (MACS Quant; Miltenyi Biotec, Bergisch Gladbach, Germany).

### 2.6. Cell Differentiation

Cells isolated from iBTA tissues were cultivated in DMEM-LG (Wako) containing 10% FBS (Biosera). When the culture nearly reached confluence, the cells were detached using trypsin-EDTA (Wako) and collected. Collected cells (0.1 mL, 1 × 10^5^ cells) were seeded into 96-well PrimeSurface plates (Sumitomo Bakelite, Tokyo, Japan) and cultivated for 3 weeks in chondrogenic and osteogenic differentiation media (Promocell, Heidelberg, Germany). After the cultivation period, the cultured spheroids were fixed in 4% paraformaldehyde and PBS (Wako), embedded in paraffin, and sectioned into 3–5-µm-thick sections. The sections were stained with Alcian blue to assess chondrocytes and von Kossa for osteocytes.

## 3. Results

### 3.1. Tissue Formation in the Wired Molds

Molds, in which six stainless steel wires with 1-mm diameters were arranged in a cylindrical shape at 4-mm intervals (Figure 1a), were embedded under the skin of beagles. When the air inside the mold was removed to bring the mold into close contact with the subcutaneous tissue, the inside was rapidly filled with body fluid. The mold was small and could be easily removed from the body through the wound at the time of embedding. The surrounding subcutaneous tissues migrated inside the mold after 0.5 weeks of embedding (Figure 1a). Most of the tissues inside the mold were fragile fibrin and adipose tissues, with few collagen fibers observed around the wires (Figure 1b). As the embedding period increased, the adipose tissue was replaced by collagen fibers between the wires (Figure 1b). After 2.5 weeks of embedding, strong tissues with minimal inflammation were formed that could be plucked (Figure 1c). The strength of the resulting tissue was not measured. However, their structure was strong enough to be lifted with a finger, as shown in Figure 1c. The internal structure was divided into a whitish, fragile area between the wires and a strong, star-shaped red area extending from the core toward the wires (Figure 1c). The segregated structure became more pronounced after 3 weeks of embedding (Figure 1d). This formed a stellate central core that occupied most of the tissue and pocket areas that filled its protrusions, which remained approximately the same at 5 weeks (Figure 1b). The entire tissue was free of elastin and mostly occupied by collagen fibers (Figure 1d). Few inflammatory cells were observed in the tissues. When the Sirius Red-stained specimen of the obtained tissue was observed using a polarizing microscope, most of the collagen was type I (yellow-colored fibers); however, the pocket area contained a large amount of type III collagen (green-colored fibers) (Figure 1e).

Two types of molds were prepared in which the wire spacing was changed stepwise (Figure 2a) or continuously (Figure 2b). The stepwise type, with an 11-mm diameter, changed the wire spacing to 3, 4, and 6 mm, and the continuous type, with a 16-mm diameter, changed the wire spacing from 1 to 8 mm. The subcutaneous embedding of both molds in the beagles filled the inside of the wire cage with tissue for 3 weeks. In the circumferential cross-section of the resulting tissue, a central star-shaped core structure and an arcuate pocket structure between all the adjacent wires were observed (Figure 2a,b). In the stepwise type, the cross-sectional area of the entire structure decreased as the wire spacing increased. When the wire spacing was wide (6 mm), the widths of the pockets were large, but their depths (approximately 2 mm) were almost the same as when the wire spacing was narrow (3 mm) (Figure 2a). In contrast, almost no pockets were observed at wire spacings less than approximately 2.5 mm in the continuous type (Figure 2b). When the wire spacing was wider than 3.5 mm, the pocket depth was approximately 4 mm.

### 3.2. Components of the Tissues Formed

**Figure 3:**
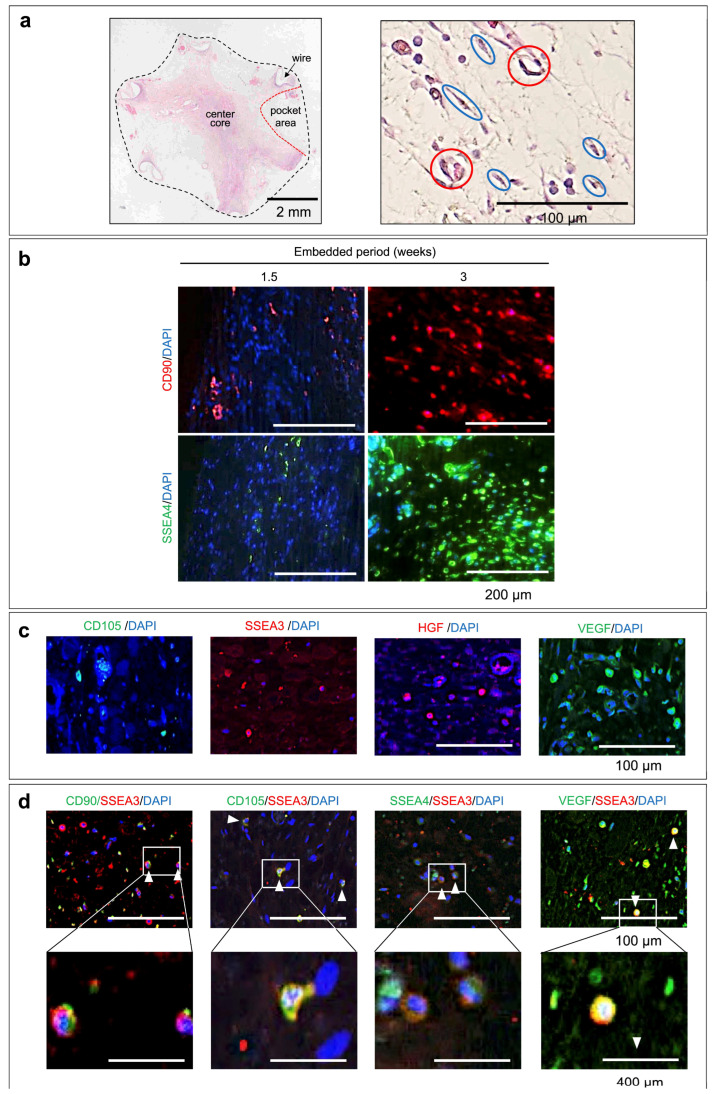
(**a**) Microscopic cross-sectional image of the whole tissue (**left**) and the pocket area (**right**) formed inside the six-wired mold (Figure 1a) after 3 weeks of embedding. In the right photo, blue circles indicate fibroblast-like cells, and red circles indicate capillary vessels in the pocket area. (**b**) Immunofluorescence images of CD90 or SSEA4 at the pocket area (**left** in (**a**)) of the tissue formed by 1.5 or 3-week mold embedding. (**c**) Immunofluorescence images of CD105, SSEA3, HGF, or VEGF at the pocket area (**left** in (**a**)) of the tissue formed by 3-week mold embedding. (**d**) Immunofluorescence double stained cells (arrows) of CD90, CD105, or SSEA4 against SSEA3 in the pocket area (**left** of (**a**)) formed by 3-week mold embedding (**top row**) and enlarged images of each square (**lower row**).

Most of the cells in the center of the tissue formed from the six-wired mold (Figure 1a) were fibroblast-like spindle-shaped cells, regardless of the implantation period. On the other hand, in addition to fibroblast-like cells, capillary vessels were observed in pocket areas around the tissue periphery (Figure 3a). Few inflammatory cells, including colony formation, were observed in the entire tissue. Interestingly, cells expressing CD90, a typical MSC marker, and SSEA4, a pluripotent stem cell marker, were observed in the pocket area around the tissue 1.5 weeks after implantation (Figure 3b). Three weeks after implantation, CD90 and SSEA4-positive cells were abundantly distributed throughout the pocket area (Figure 3b). In addition, cells expressing other MSC and pluripotent cell markers such as CD105 and SSEA3 were also observed in the same pocket region (Figure 3c). Furthermore, growth factors such as hepatocyte growth factor (HGF) and vascular endothelial growth factor (VEGF) were also expressed (Figure 3c). Upon immunohistochemical double staining, SSEA3-positive stem cells expressed other stem cell markers, such as CD90, CD105, and SSEA4 (Figure 3d).

**Figure 4:**
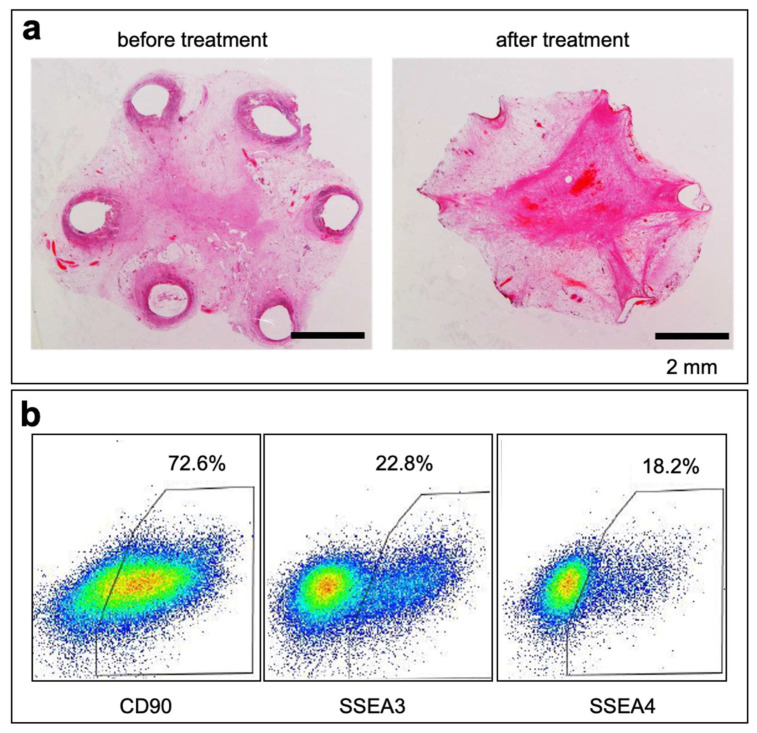
(**a**) Microscopic cross-sectional photos of the tissues obtained after 3 weeks of embedding the six-wired molds (Figure 1a) before and after collagenase treatment. (**b**) Flow cytometric analyses of cells isolated using collagenase treatment. Cells were stained with CD90, SSEA3, and SSEA4.

A collagenase treatment was performed to isolate the cells from the formed tissues. Upon treatment, only the peripheral areas, including stem cell-rich tissues, were selectively degraded (Figure 4a). Approximately four million cells were isolated from a single mold. The flow cytometry analysis revealed the proportion of stem cell marker-positive cells. Approximately 70% of the cells expressed the CD90 antigen. The pluripotent stem cell markers SSEA3 and SSEA4 were present in approximately 20% of the cells (Figure 4b).

### 3.3. Differentiation of Isolated Cells

**Figure 5:**
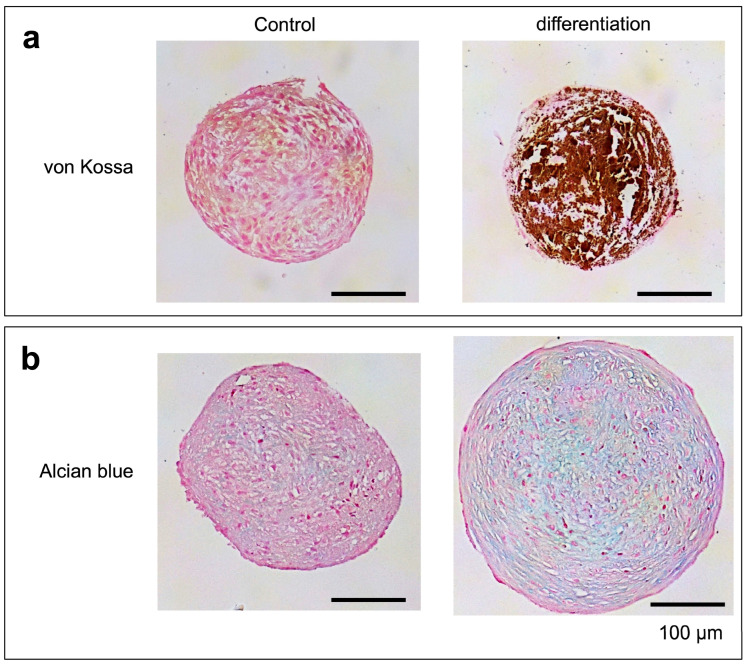
Differentiation of isolated cells from tissues obtained after 3 weeks of embedding the six-wired molds (Figure 1a). (**a**) Histological von Kossa stains after osteogenic culture. (**b**) Histological Alcian blue stains after chondrogenic culture.

To preliminarily evaluate the differentiation potential of the isolated cells, general cartilage or bone differentiation cultures were performed in spheroids. Calcium accumulated inside the bone-forming differentiated spheroids, as determined by von Kossa staining (Figure 5a). Spheroids cultured for cartilage differentiation expanded more than the control spheroids and accumulated proteoglycans (Figure 5b).

## 4. Discussion

In this study, we developed a unique device to efficiently collect stem cells in the body. The greatest achievement of this study was the successful collection of large numbers of stem cells expressing pluripotency markers (SSEA3 or SSEA4). SSEA3 and SSEA4 are localized on the cell surface of human EC or ES cells, et. al., and are used as pluripotency/embryonic markers. Since SSEA3 and SSEA4 positive cells are likely to be pluripotent stem cells, the positive cells were isolated from the formed tissues and examined for their ability to differentiate into cartilage and bone tissues as a preliminary confirmation. Methods for efficiently harvesting large numbers of MSCs have already been established [23,24]. On the other hand, very few pluripotent stem cells exist in the body, and the abundance of pluripotent stem cells is extremely low, even in bone marrow. Our method is simple and feasible, as it embeds the molds under the skin. Previously, we found that somatic stem cells were present in the outer regions of biotubes with 1-mm thick walls formed using tubular molds [22]. These stem cells were primitive, as they expressed SSEA4, an embryonic stem cell marker. Primitive stem cells can be obtained using the iBTA technology. Other research groups have also reported the presence of stem cells in foreign body reactions [16,17]. Here, we clarified that stem cells, including SSEA3- or SSEA4-positive cells, could be actively recruited simply by creating an artificial space in the body by using the mold. Because the developed device was small, it could be easily harvested from the wound at the time of implantation. The mold could be easily disassembled, and the formed tissue could be removed without causing any damage. The obtained tissue contained not only stem cells but also growth factors, so it was thought that it could be used as a medical material in its original state.

The tissue structures formed in the molds differed depending on the wire spacing. When the wire spacing was narrow (<2 mm), the molds were filled with tight connective tissue; however, when the spacing was wide, the formed tissue was sparse. Low-density tissue formed shallowly where the wires were narrower and deeply (at approximately 4 mm) where the wires were wider. The analysis of the structures formed in the molds after implantation revealed changes over time. Immediately after implantation, the insides of the molds were mainly filled with body fluid and fibrin, and almost no collagen was present. After a few days, subcutaneous fibroblasts migrated, and an encapsulation reaction occurred around the wires, thus forming collagen tissue. Collagen was then produced over time, and the insides of the molds were filled with connective tissue. Blood vessels are formed when surrounding cells produce growth factors that supply progenitor cells and the nutritional factors necessary for tissue formation. In this process, a large number of highly activated progenitor cells are needed in areas where tissue formation is in progress (i.e., immature tissue between wires), and many stem cells are expected to be mobilized. Pluripotent stem cells are thought to contribute to tissue formation by supplying the nutritional factors necessary for tissue formation and by differentiating into cells that compose the tissue. The formation of space under the skin and the induction of large-scale tissue formation compared to that of normal tissue resulted in the accumulation of many highly active stem cells expressing pluripotent stem cell markers (Figure 5).

MSCs are currently the most widely used stem cells for clinical applications. Stem cells have various functions, including the ability to differentiate into various tissue-specific cells such as bone, cartilage, and fat. They can further regulate immunity and produce growth factors. Although MSCs can be isolated from various tissues, such as bone marrow and adipose tissue, the presence of MSCs is low, and their properties vary depending on the tissue from which they are derived. Interestingly, the stem cells obtained in this study expressed SSEA3 and SSEA4, which are markers expressed by pluripotent stem cells, such as ES and iPS cells [2,3]. Multilineage-differentiating stress-enduring (Muse) cells, which are somatic stem cells, express SSEA3 and CD105 and have attracted attention as a cell source for regenerative medicine because of their pluripotency and non-tumorigenicity [25,26]. In this study, some of the accumulated cells in the molds expressed these markers, suggesting the presence of cells similar to Muse cells. The proportion of Muse cells in adult bodies is very low (1/3000 of bone marrow cells). Many of the SSEA3-positive cells in this study accumulated in immature tissues between the columns of the molds. When the obtained tissues were degraded by collagenase, only the peripheries, which are rich in stem cells, were degraded by treatment with 0.25% collagenase for 1.5 h. Thereafter, approximately four million cells were obtained. Although the enzyme treatment conditions were not optimized in this study, the stem cells could be efficiently recovered by selectively degrading the crescent-shaped areas between wires. Because the proportion of stem cells in the adult body is generally low, this method provides an innovative way to collect a high density of stem cells. The stem cells obtained using this method had the ability to differentiate into osteocytes and chondrocytes, which is a characteristic of MSCs [27].

Stem cell therapy involves the administration of cells on the order of 10^6^ to 10^7^ cells. That is, the number of cells that could be obtained without culture from one developed device was 4 × 10^6^ cells, which was a sufficient amount to be used for that treatment. Regarding MSCs, the number of MSCs contained in bone marrow is very low [11]. Approximately 5 × 10^3^ ASCs can be collected from 1g of adipose tissue [11]. In therapy, ASCs isolated from several grams of adipose tissue are cultured for several weeks to reach a level of 10^6^ to 10^7^ cells [23]. If we were to obtain 4 million MSCs from the body without culturing, we would need to harvest 800 g of fat. In the case of 10^7^ cells, 2 kg of fat must be collected, which is unrealistic. Furthermore, it is astonishing that among the 4 million cells collected, 20%, or 800,000 cells, were SSEA3 or SSEA4 positive. There are few reports of the successful development of instruments to collect SSEA3 or SSEA4-positive cells. MUSE cells are isolated from MSCs harvested from bone marrow, skin, fat, etc., so the number of cells obtained is even smaller. Therefore, culturing MUSE cells is essential for clinical application. MUSE cells have been shown to be effective in subacute ischemic strokes in clinical trials [28]. On the other hand, similar to the developed device, it has been shown that biosheet tissues containing pluripotent stem cells formed in the body were effective for wound closure when applied to a diabetic ulcer immediately after being collected from the body [29]. The present study represents a breakthrough in the accumulation of stem cells under the skin and may be applied to cell therapies using autologous stem cells. This method has the potential to solve the qualitative and quantitative problems associated with conventional cell therapies.

The obtained collagenous tissues contained growth factors, especially VEGF. Blood vessel formation is important for tissue formation. It has been reported that cells that accumulate in foreign body reactions contribute to the formation and stabilization of blood vessels. In the study, subcutaneous spaces were created by placing molds under the skin, and neoplastic tissues were formed in these spaces. During neoplastic tissue formation, blood vessels are necessary to supply essential cells and nutrients. Stem cells are thought to contribute to tissue and blood vessel formation through their differentiation ability and angiogenesis. As iBTA induces tissue formation in subcutaneously created space, tissue formation occurs on a larger scale than in normal tissue repair or encapsulation reactions. Therefore, stem cells with high tissue repair activity may accumulate. The ability to produce growth factors affects the efficacy of cell therapy. The highly active stem cells obtained using this method may be useful cell sources for effective cell therapy. Other devices based on iBTA technology are also being developed in separate studies. The tubular tissue obtained with this device has already been used as Biotube regenerated blood vessel for patients with severe lower limb ischemia or dialysis [30,31]. All patients did not require hospitalization during the device embedding period, which lasted several weeks for Biosheet preparation and several months for a biotube and went about their daily lives with little discomfort. Adhesion between the device and subcutaneous tissue was mild, and its harvest was smooth. Although subcutaneous hematoma occurred on a few occasions after device extraction, none of the extractions caused severe trauma associated with pain syndromes. iBTA-based therapy is a safe medical technology. There, the resulting stem cell-containing tissue was applied to the wound in its original state. We are also considering using the tissue containing stem cells obtained in this research for treatment without separating the cells.

In this study, we demonstrated the presence of cells expressing pluripotent stem cell markers in tissues formed by iBTA and developed molds for their collection. This innovative method simply implants a mold to maintain space under the skin and obtains stem cells that express pluripotent stem cell markers and have the ability to multipotently differentiate. With iBTA, it is possible to obtain a large number of stem cells without the need for a cell culture process. iBTA may therefore be applied to therapeutic applications at a low cost. Future research may lead to the functional analysis of cells and their therapeutic applications.

## 5. Conclusions

**Figure 6:**
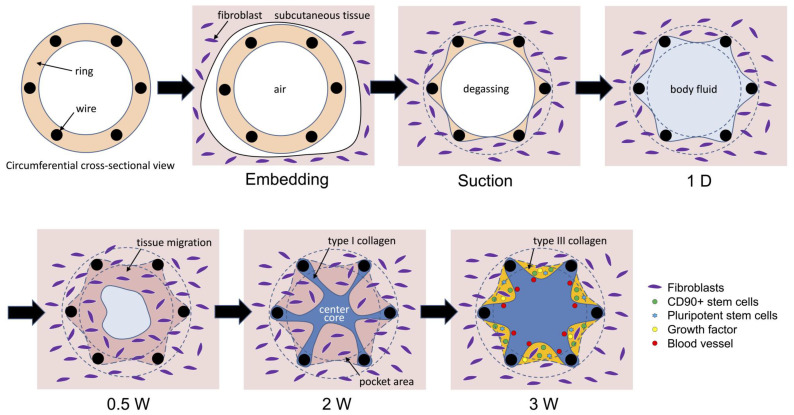
Tissue formation process using the six-wired molds shown in Figure 1a.

We have successfully developed a device that can form collagen-based tissues in the body that efficiently collect cells positive for the pluripotent stem cell markers SSEA3 or SSEA4 (Figure 6). The resulting tissue also contains growth factors such as HGF and VEGF, so it is expected to be effective as a therapeutic material. iBTA does not require an in vitro cell culture process and can obtain a large number of autologous stem cells in a fresh state, so it has the potential for low-cost therapeutic applications. We aim to contribute to the use of stem cell-containing tissues as medical materials and cell therapy in regenerative medicine.

## Data Availability

The authors declare that the data supporting the findings of this study are available within the paper.
